# Reelin Counteracts Chondroitin Sulfate Proteoglycan-Mediated Cortical Dendrite Growth Inhibition

**DOI:** 10.1523/ENEURO.0168-20.2020

**Published:** 2020-07-28

**Authors:** Eric Zluhan, Joshua Enck, Russell T. Matthews, Eric C. Olson

**Affiliations:** 1 Department of Neuroscience and Physiology, State University of New York Upstate Medical University, Syracuse, NY 13210; 2 Developmental Exposure to Alcohol Research Center (DEARC), Binghamton University, Binghamton, NY 13902

**Keywords:** CSPG, Dab1, dendritogenesis, lissencephaly, Reelin

## Abstract

Disruptions in neuronal dendrite development alter brain circuitry and are associated with debilitating neurological disorders. Nascent apical dendrites of cortical excitatory neurons project into the marginal zone (MZ), a cell-sparse layer characterized by intense chondroitin sulfate proteoglycan (CSPG) expression. Paradoxically, CSPGs are known to broadly inhibit neurite growth and regeneration. This raises the possibility that the growing apical dendrite is somehow insensitive to CSPG-mediated neurite growth inhibition. To test this, developing cortical neurons were challenged with both soluble CSPGs and CSPG-positive stripe substrates *in vitro*. Soluble CSPGs inhibited dendritic growth and cortical dendrites respected CSPG stripe boundaries, effects that could be counteracted by prior CSPG inactivation by chondroitinase. Importantly, addition of Reelin, an extracellular signaling protein highly expressed in the MZ, partially rescued dendritic growth in the presence of CSPGs. High-resolution confocal imaging revealed that the CSPG-enriched areas of the MZ spatially correspond with the areas of reduced dendritic density in the Reelin null (*reeler*) cortex compared with controls. Chondroitinase injections into *reeler* explants resulted in increased dendritic growth into the MZ, recovering to near wild-type levels. Activation of the serine threonine kinase Akt is required for Reelin-dependent dendritic growth and we find that CSPGs induce Akt dephosphorylation, an effect that can be counteracted by Reelin addition. In contrast, CSPG application had no effect on the cytoplasmic adaptor Dab1, which is rapidly phosphorylated in response to Reelin and is upstream of Akt. These findings suggest CSPGs do inhibit cortical dendritic growth, but this effect can be counteracted by Reelin signaling.

## Visual Abstract

**Figure:**
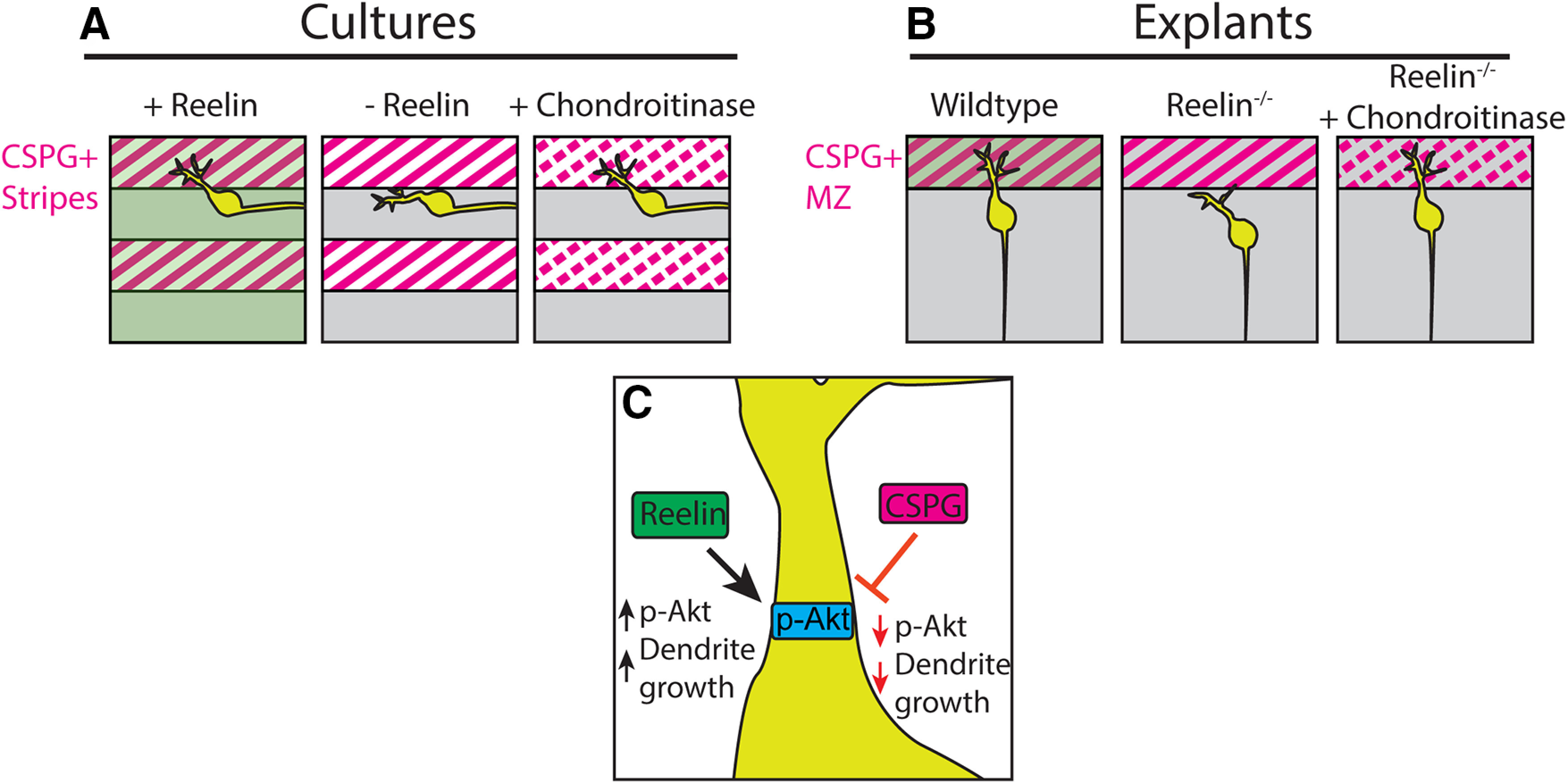

## Significance Statement

Appropriate dendritic development is essential for normal neuronal function throughout life. The area where most cortical dendrites initially project (the marginal zone) is cell sparse but highly enriched in chondroitin sulfate proteoglycans (CSPGs). While CSPGs are known to inhibit axonal outgrowth, their impact on dendritic growth is unclear. This study demonstrates that the growth of the apical dendrite is also inhibited by CSPGs. However, this inhibitory effect can be reversed by chondroitinase treatment and by activation of the Reelin signaling pathway. Disruptions in Reelin signaling cause intellectual disability and have been linked to autism. Thus, these findings identify a context in which Reelin signaling operates and provide insight into the underlying mechanism of neurodevelopmental disorders.

## Introduction

The structure of neuronal dendrites is an identifying feature of neuron subtypes and determines neuronal responses to synaptic input. Dendritic structural abnormalities are associated with both neurodevelopmental disease and neurodegeneration (Kulkarni and Firestein, 2012). Precise integration of both intrinsic factors and extrinsic factors is crucial for the formation of the dendritic arbor (McAllister, 2002; Jan and Jan, 2003; Valnegri et al., 2015). The forming apical dendrites of cortical excitatory neurons project into the marginal zone (MZ) as the neurons complete migration (Pinto-Lord et al., 1982; Olson et al., 2006; O'Dell et al., 2015).

The MZ is enriched in Reelin, an extracellular protein secreted by Cajal–Retzius neurons that are located in the MZ. While the Reelin null (*reeler*) cerebral cortex is well known for its characteristically disorganized and approximately inverted cellular layering, deficiency in Reelin signaling also causes a dramatic dendritic disruption with stunted, misoriented dendritic growth both *in vivo* (Pinto Lord and Caviness, 1979; Niu et al., 2004; Olson et al., 2006; Nichols and Olson, 2010) and *in vitro* (Niu et al., 2004; Jossin and Goffinet, 2007). Prior time-lapse imaging of dendritic initiation and growth showed that, in the *reeler* cortex, a subset of neurons demonstrate appropriate dendritic initiation and early projection into the MZ. However, in contrast to controls, the *reeler* dendrites become unstable and retract from the MZ, reorganizing tangentially underneath the MZ. Application of recombinant Reelin protein to *reeler* cortices caused the rapid re-projection of the dendrite into the MZ (O'Dell et al., 2015). These findings suggest a context-specific function for Reelin in stabilizing the nascent apical dendrite in the MZ and the potential existence of a dendrite de-stabilizing factor in the MZ that exerts its effect in the absence of Reelin signaling.

During development the neural extracellular matrix (nECM) in the MZ is characterized by high expression levels of chondroitin sulfate proteoglycans (CSPGs; Sheppard et al., 1991; Meyer-Puttlitz et al., 1996). CSPGs are composed of core proteins modified with highly sulfated glycosaminoglycan side chains (Bandtlow and Zimmermann, 2000). CSPGs are generally inhibitory to neurite outgrowth, specifically axonal outgrowth and axon regeneration (Sharma et al., 2012). CSPGs may inhibit neurite growth by acting as an anti-adhesive substrate (Emerling and Lander, 1996), but specific signaling events have also been demonstrated. For example, subsets of CSPGs are known to bind and augment the signaling of other compounds in the nECM (Smock and Meijers, 2018). In addition, some CSPGs have been shown to directly bind and activate receptors such as receptor tyrosine phosphatase σ (RPTPσ) and leukocyte common antigen-related phosphatase (LAR), which are known to functionally inhibit axonal regeneration (Fisher et al., 2011; Lang et al., 2015).

Dendritic growth into the CSPG-rich MZ in wild-type cortices suggests that the growing apical dendrite may be insensitive to CSPG-mediate neurite growth inhibition. However, the observation that in *reeler* cortices dendrites avoid the MZ raises the possibility that CSPGs are inhibitory to dendritic outgrowth and Reelin signaling may counteract this inhibition. To test the effects of CSPGs on dendritic growth, embryonic cortical neurons were cultured in the presence of purified neural CSPGs presented either in soluble form or as patterned stripe substrates. In these assays, CSPGs were inhibitory to cortical dendritic growth and branching. This inhibition could be counteracted by inactivating the CSPGs with chondroitinase ABC (chABC), an enzyme that abrogates CSPG binding to its receptors, or partially counteracted by the application of recombinant Reelin protein.

We further addressed the possibility that CSPGs destabilize the apical dendrite in the absence of Reelin using a whole hemisphere explant approach. This organotypic explant captures early cortical development [embryonic day (E)13–E15], including normal preplate splitting, a Reelin signaling-dependent process (Nichols and Olson, 2010). We show that digestion of CSPG side chains with chondroitinase partially rescued dendritic projection patterns within the MZ of *reeler* explants. We also found that the serine threonine kinase Akt, a common downstream signaling component for both Reelin signaling and some CSPG signaling pathways was reciprocally regulated by CSPGs and Reelin, with Reelin signaling counteracting CSPG-mediated Akt inactivation. These findings suggest that an important function of Reelin signaling is to counteract CSPG-mediated dendrite destabilization.

## Materials and Methods

### Mice

Animals were used in compliance with approved protocols by the Institutional Animal Care and Use Committee. *Reeler* (B6C3Fe a/a-Reln; Jackson Laboratories) and *scrambler* (A/A-Dab1 scm/J; The Jackson Laboratory) heterozygote mice were mated to produce Reelin-deficient *reeler* and Dab1-deficient *scrambler* mutant embryos, respectively. For primary culture experiments (except *scrambler* cultures), embryonic cortices from *reeler* heterozygote matings were pooled together before dissociation. Cortices from *scrambler* litters were genotyped and dissociated separately. For explant injection experiments, the embryos were genotyped after explant preparation. The day of plug discovery is considered E0. Sexes were combined for all experimental analyses.

### Explants cultures

Whole hemisphere explants were prepared at E13 (Nichols et al., 2013). Embryonic cerebral ventricles were injected with 0.8 mg/ml doublecortin (DCX)-mRFP (Wang et al., 2007) plasmid DNA into the cortical ventricle and electroporated, *ex utero*. Explants were cultured medial side down on 3-μm pore size collagen-coated polytetrafluoroethylene filters (Transwell-COL) in DMEM-F12 medium + GlutaMAX and supplemented with 2% B27, 1% G5, and 1× penicillin (all from Invitrogen). The explants were maintained in a high oxygen Billups-Rothenberg Incubator chamber within a standard 37°C tissue culture incubator. Two days later, the explants were either taken for primary cultures or used for histology.

### Primary neuron cultures

Dorsal regions of neocortex were dissected from embryos at E15 or from explants 2 d *in vitro* (DIV) after E13 *ex utero* electroporation and culturing. The tissue was dissociated in 0.25% Trypsin-EDTA for 15 min in a 37°C water bath with occasional, gentle trituration. The dissociated cells were then plated in Neurobasal medium supplemented with 2% B27, 1× GlutaMAX, and 1× Pen/Strep on 24- or 96-well plates coated with 50 μg/ml poly-D-lysine (Sigma). Cultures were seeded at 1.2 × 10^6^ cells/well (24-well plate) for Western blottings, 1 × 10^5^ cells/well of a 96-well plate for subsequent microscopic analyses of morphology, or 2.5 × 10^4^ cells/well of a 96-well plate for quantitative immunocytochemistry. The cultures were maintained in a 37°C, 5% CO_2_ incubator. After 2 DIV (E17 equivalent), cultures were either fixed for microscopic analyses or scraped and lysed for Western blot analyses. For exogenous CSPG exposure, purified neural CSPGs (CC117, EMD Millipore) were suspended in PBS at 50 μg/ml and added to the medium of cultured neurons at 3 μg/ml.

### Stripe assay

CSPG stripes (50 μg/ml) were patterned on PEI-coated 50-mm dishes with silicon matrices by overnight incubation at 37°C (Knöll et al., 2007). The next day, the matrices were removed, and the stripe area was immediately coated with Matrigel (354277, Corning) for 1 h. Dissociated cortical neurons were then added to the stripes. After 24 h, Reelin or control media was added to the cultures for an additional 24 h and subsequently fixed in paraformaldehyde (PFA).

### chABC injection

Ten-microliter Wiretrol glass pipettes (Drummond Scientific Co) were pulled to a fine point using a Sutter P-87 Microelectrode Puller (Sutter Instruments), and the tip was broken with jeweler’s forceps. On E15, chABC (100 mU/μl; C3667, Sigma) or PBS injections (∼1 μl) were made at three to four points along the dorsolateral area of the explant, corresponding to dorsomedial cortex. Injection periods were brief (<15 min) and conducted at room temperature under a dissection microscope (Olympus SZX12). The explants were then returned to the high-oxygen environment and cultured for an additional 16 h.

### Quantification of dendritic projection into the MZ

The amount of dendritic projection within the MZ in different experimental conditions was approximated as described (O'Dell, 2012, 2015) with minor modifications. Z-series were flattened and thresholded using NIH ImageJ (W. Rasband, National Institutes of Health, Bethesda, MD). The integrated density of mRFP-positive pixels in the MZ, defined as the area within 15 μm of the pial surface, was normalized to the integrated density of mRFP-positive pixels in underlying 35 μm of cortical plate (CP). This latter value roughly reflects the number of mRFP-positive electroporated neurons contributing mRFP-positive dendrites to the overlying MZ. The mRFP-positive MZ/mRFP-positive CP ratios then become an approximation of dendritic projection in the MZ per neuron. These values were then compared between experimental conditions.

### Conditioned media production

A HEK293 cell line was used to produce control-conditioned media (CM) and a stably transfected HEK293 cell line (Michael Frotscher, University of Freiburg) was used to produce Reelin-conditioned media (RM). Cells were cultured to near-confluence, washed in Opti-MEM media and incubated for 48 h in serum free Opti-MEM media supplemented with 1× GlutaMAX and 1× Pen/Strep. Centrifugation of the conditioned media in Amicon Ultra 100,000 kDa molecular weight cut off filters (Millipore) brought the medias to a 10× concentration, and medias were diluted fivefold on addition to cultures (Nichols and Olson, 2010).

### Immunocytochemistry

Cells were fixed in 4% PFA in Pagano solution (250 mm sucrose, 25 mm MgCl_2_, 2.5 mm KCl, 25 mm HEPES, pH 7.4; + phosphatase inhibitors for phospho immunostaining) for 15 min at room temperature, rinsed 3× for 5 min in Pagano, and incubated with blocking solution (Pagano + 0.25% Saponin + 2% BSA) for 1 h. Cells were incubated in primary antibodies diluted in blocking solution for 1 h, rinsed 3× for 5 min with Pagano solution, incubated with appropriate secondary for 1 h, then rinsed 3× for 5 min with Pagano solution. The primary antibodies used were anti-MAP2 (1:250, Chemicon, ab5756), anti-SMI-312 (1:1000, Covance, SMI312R), anti-p-Akt (1:100, Santa Cruz, sc-514032), Cat-315 (1:20, from R. Matthews; Dino et al., 2006), CS56 (1:200, Abcam, ab11570), and anti-DCX (1:500, gift from C. Walsh; Gleeson et al., 1999) polyclonal sera. Appropriate Alexa Fluor 488-conjugated, Alexa Fluor 555-conjugated, and Alexa Fluor 647-conjugated secondary antibodies were used. Hoechst 33342 (2 μg/ml; Invitrogen) was used to counterstain nuclei. Immunohistochemistry for explants was performed as previously described (Nichols et al., 2013). Images were collected with a Zeiss LSM780 laser scanning confocal microscope (SUNY Upstate Advanced Fluorescence Imaging Core).

### Western blot analysis

Cells were rinsed in ice-cold PBS then lysed in ice-cold RIPA buffer (50 mm Tris-HCl, 1% NP-40, 0.25% Na-deoxycholate, 150 mm NaCl, and 1 mm EDTA, pH 7.4) supplemented with phosphatase inhibitors (1.0 mm sodium orthovanadate and 20 mm sodium fluoride) and a protease inhibitor mixture (P-8340, Sigma). All samples were loaded into a 10% SDS-PAGE gel and transferred to a 0.45-μm PVDF Immobilon-FL membrane (EMD Millipore). Membranes were incubated for 1 h at room temperature in Odyssey blocking buffer (LI-COR Biosciences) then incubated with primary antibodies overnight at 4°C. The primary antibodies were p-Akt (mouse 1:1000, Santa Cruz, sc-514032), Akt (1:1000, Cell Signaling, 4685). p-ERK1/2 (1:000, Santa Cruz, sc-7383), anti-GAPDH (1:2000, UBPBio, Y1041), anti-pY99 for detection of phosphorylated Dab1 (1:1000, Santa Cruz, sc-7020), and anti-Dab1 (1:1000, Sigma, AB5840). Appropriate IRDye 800CW and IRDye 680RD secondary antibodies (LI-COR Biosciences) were used and membranes were scanned on an Odyssey CLx system (LI-COR Biosciences).

### Statistical analyses

Data in graphs are shown as mean ± SEM. Experiments comparing a single determination of means between two independent groups were analyzed with Student’s *t* test. One-way ANOVA with Šidák multiple comparison *post hoc* tests were used for comparisons between multiple means. Data organization was conducted in Microsoft Excel and statistical analyses were conducted in GraphPad Prism 8.

## Results

### CSPG inhibition of cortical dendrite growth is rescued by Reelin addition *in vitro*

**Figure 1.**
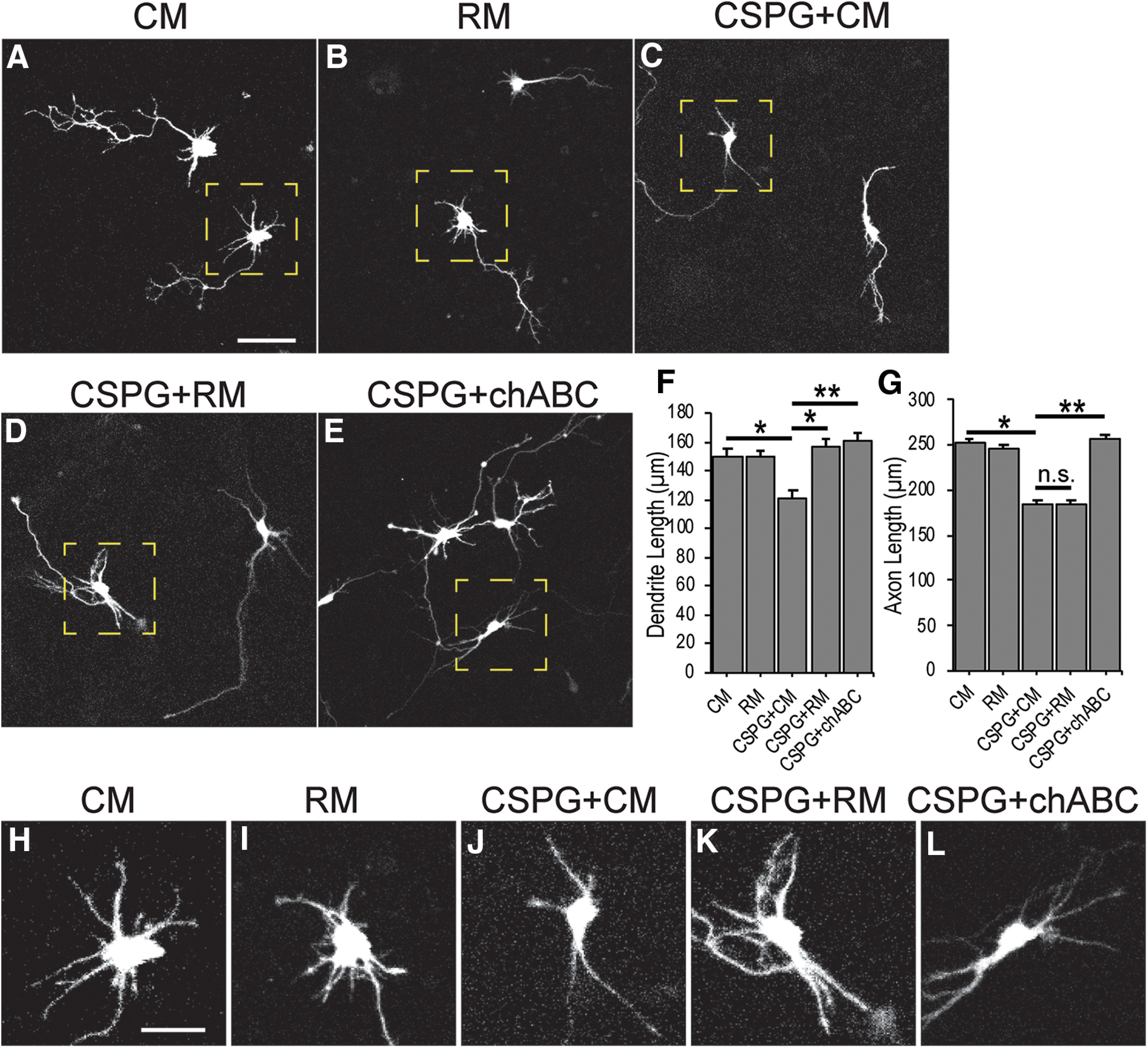
Reelin counteracts CSPG-mediated inhibition of dendritic outgrowth. Primary cortical neurons were fluorescently labeled by electroporation of a DCX-mRFP expression plasmid that drives red fluorescent protein expression in immature neurons. After 1 DIV, neurons were challenged with (***A***, ***H***) conditioned media (CM), (***B***, ***I***) Reelin-conditioned media (RM), (***C***, ***J***) purified CSPGs (3 μg/ml) + CM, (***D***, ***K***) CSPGs+RM, or (***E***, ***L***) chondroitinase pretreated CSPGs and then fixed after one additional DIV. ***F***, Quantification after neurite tracing revealed that CSPG+CM significantly reduced total dendritic length, an effect that could be reversed by RM or chondroitinase (chABC) pretreatment. ***G***, In contrast, axonal length reduction by CSPG could not be reversed by RM. ***H–L***, Cropped images of representative dendritic processes in yellow dashed boxes in ***A–E***, respectively. *N* ≥ 30 neurons in each condition; *, different at *p* < 0.05; **, different at *p* < 0.01. One-way ANOVA with Šídák’s multiple comparisons *post hoc* test. Not significant (n.s.). Scale bars = 25 μm (***A***) and 10 μm (***H***).

Although CSPGs often inhibit neurite outgrowth, their effects can vary depending on the context (Wu et al., 2004; Dutt et al., 2011). Reelin’s effects also appear to be context specific. For instance, Reelin signaling has a robust role in dendritogenesis *in vivo* (Pinto Lord and Caviness, 1979; Olson et al., 2006; O'Dell et al., 2012, 2015), but Reelin’s dendritic growth effects *in vitro* are smaller and have not been described in cortical neurons (Niu et al., 2004; MacLaurin et al., 2007). To expressly test acute effects of CSPGs and Reelin in control of cortical dendrite growth, we prepared cortical neurons for *in vitro* testing. Developing cortical neurons were labeled in explants by electroporation of a DCX-mRFP expression construct (Wang et al., 2007) on E13 to sparsely label neurons for subsequent neurite tracing (Longair et al., 2011). To test whether CSPGs directly affect dendrite growth, we dissociated and cultured DCX-mRFP-positive neurons from explants 2 d after electroporation and added purified neural CSPGs containing versican, phosphocan, neurocan, and aggrecan (Millipore; 3 μg/ml) to the media. Cultures were fixed after 2 DIV, at which point labeled neurons displayed characteristic polarity with a single long axon and multiple shorter dendrites (Fig. 1*A–E*). At this early stage of differentiation (2 DIV), commonly used molecular markers do not uniquely distinguish dendrites and axons (Caceres et al., 1986; Kosik and Finch, 1987). Therefore, the axon was determined by morphology (longest thin process), and the remainder of the neurites emanating from the cell body and their branches were assigned dendritic identity (Bartlett and Banker, 1984; Dotti et al., 1988; Barnes and Polleux, 2009). Cells cultured with CSPG containing media displayed decreased dendrite length compared with control media treatment (CM = 150.0 μm vs CSPG+CM = 121.5 μm, *p* = 0.013; Fig. 1*C*,*J*,*F*), along with decreased axon length (CM = 252.2 μm vs CSPG+ CM = 184.5 μm, *p* = 0.014; Fig. 1*C*,*G*). To test whether this inhibition was because of functional CSPG interactions, we treated CSPGs with chABC, an enzyme that cleaves the chondroitin sulfate side chains from proteoglycan core proteins. Side chain removal abrogates CSPG binding to their receptor(s) and thereby functionally inactivates the CSPG (Silver and Miller, 2004; Sharma et al., 2012). Pretreating the purified CSPGs with chondroitinase prevented inhibition of both dendrites (CSPG+CM = 121.5 μm vs CSPG+chABC = 161.3 μm, *p* = 0.003; Fig. 1*E*,*F*,*L*) and axons (CSPG+CM = 184.6 μm vs CSPG+chABC = 257.2 μm, *p* = 0.009; Fig. 1*E*,*G*). Next, we tested whether CSPG inhibition *in vitro* is affected by the presence of Reelin by adding RM to the cultures. Interestingly, Reelin addition alone had no effect on dendrite growth in our experiments (CM = 150.0 μm vs RM = 149.4 μm, *p* = 0.921; Fig. 1*B*,*F*,*I*). However, Reelin addition reversed the inhibition of dendritic growth observed with CSPGs (CSPG+CM = 121.5 μm vs CSPG+RM = 156.9 μm, *p* = 0.011; Fig. 1*C,D*,*F*,*J*,*K*), while having no effect on axons (CSPG+CM = 184.6 μm vs CSPG+RM = 183.8 μm, *p* = 0.999). Our results agree with previous findings that axons are sensitive to CSPG stimulation (Wang et al., 2008) but not Reelin (Jossin and Goffinet, 2001). The findings that Reelin alone has no measurable effect on dendrites but reverses CSPG-dependent dendrite inhibition might shed light on Reelin’s context-specific effects.

### CSPG stripes inhibit dendrite growth which is rescued by Reelin or chondroitinase treatment

**Figure 2.**
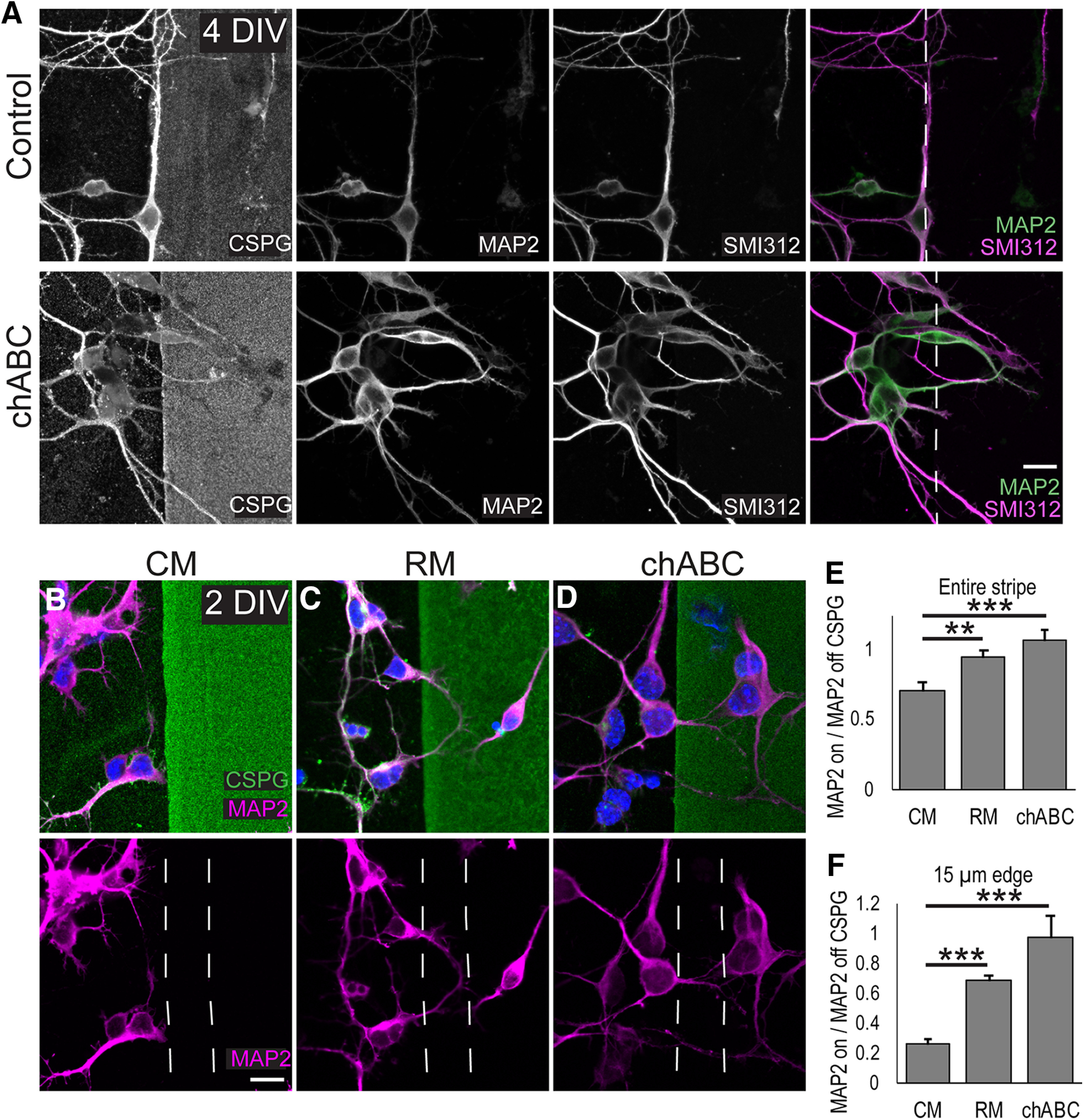
Reelin counteracts dendritic inhibition at CSPG stripe boundaries. ***A***, CSPGs inhibit dendrites and axons at CSPG-positive border. E15 primary neurons were cultured on CSPG stripes (upper panels) or CSPG stripes treated with chondroitinase (lower panels). At 4 DIV, cultures were fixed and immunostained for MAP2 to identify dendrites, SMI-312 to identify axons, and Cat-315 to identify the CSPG stripes. Chondroitinase treatment permitted growth of neurites onto the CSPG stripes. ***B–F***, To determine short term effects of Reelin on dendrites, neurons were cultured on CSPG striped substrate for 1 DIV and then stimulated with (***B***) CM or (***C***) RM for 24 h. ***D***, In parallel cultures, CSPGs were pretreated with chABC. At 2 DIV, cultures were fixed and immunostained for MAP2 to identify neurites and for CSPG to identify stripe areas. ***E***, MAP2-positive signal on the entire width of the CSPG-positive stripe was normalized MAP2-positive on CSPG-negative stripes. ***F***, MAP2-positive signal in 15-μm-wide areas at the stripe edge. The dashed lines indicate the areas of CSPG-positive stripes that were used to quantify MAP2-positive signal that was then normalized to the MAP2 signal on the adjacent 15 μm on the CSPG-negative stripe. *N* = 20 regions quantified in each condition; **, different at *p* < 0.01; ***, different at *p* < 0.001, Student’s *t* test. Scale bar = 10 μm.

Inhibition of developing axons in response to CSPG substrates has been well documented (Walter et al., 1990; Snow, 1994), but the response of embryonic cortical neuron dendrites to localized CSPG substrates is not known. Because the MZ contains localized expression of CSPGs, we reasoned that a stripe assay (Walter et al., 1987a; Knöll et al., 2007) with alternating CSPG-positive and CSPG-negative stripes might mimic the effects of cortical dendrites encountering the MZ *in vivo*. To test the effects of localized CSPGs on cortical dendrites, we generated CSPG-positive or chondroitinase-treated CSPG-positive stripes and plated E15 embryonic cortical neurons on the substrates. CSPG-positive stripes were identified with a Cat-315 antibody that binds a carbohydrate epitope on a subset of CSPGs that is not sensitive to digestion with chondroitinase (Matthews et al., 2002; Dwyer et al., 2012, 2015) or CS56 which detects chondroitin sulfate side chain epitopes (Avnur and Geiger, 1984). After 4 DIV, neurons have undergone extensive dendritic and axonal outgrowth. Neurons were immunolabeled with an antibody directed against the dendrite marker MAP2 and axonal marker SMI-312. However, even at 4 DIV, MAP2 and SMI-312 immunosignals did not exclusively localize to dendrites and axon, respectively. MAP2, in particular labeled the somatodendritic compartment. However, MAP2 signal did preferentially identify short dendrite-like neurites and SMI-312 preferentially labeled long axon-like neurites (Fig. 2*A*). Therefore, MAP2 and SMI-312 signal was used for analyses of dendritic and axonal growth, respectively. As expected SMI-312-positive axons avoided the CSPG-positive stripes, and similarly, MAP2-positive dendrites also avoided the CSPG-positive stripes (Fig. 2*A*). However, axons and dendrites grew indiscriminately onto chondroitinase-treated CSPG stripes. This shows, for the first time, that CSPG-enriched areas are relatively non-permissive for the growth of the nascent cortical dendrite. These finding are surprising, given that wild-type cortical dendrites grow into the CSPG-rich MZ *in vivo*.

Dendritic avoidance of CSPG stripes *in vitro* is reminiscent of the dendritic avoidance of the MZ that *reeler* dendrites exhibit *in vivo* (O'Dell et al., 2012, 2015). We therefore tested whether Reelin addition to the cultures could reverse dendritic inhibition on the CSPG-positive stripes. Because Reelin’s biochemical and cellular effects on cortical dendrites are rapid (Jossin and Goffinet, 2007; Nichols and Olson, 2010; O'Dell et al., 2015), we performed short-term applications to test for Reelin and CSPG interactions in neurons undergoing dendrite initiation. E15 embryonic cortical neurons were plated on CSPGs stripes and at 1 DIV, Reelin or control media was added to the cultures. Cultures were fixed at 2 DIV and immunostained for MAP2 and CSPG. This allowed quantification of MAP2-positive pixels on CSPG-positive stripes normalized to MAP2-positive pixels on CSPG-negative stripes (Fig. 2*B–D*). Of note, MAP2 signal was present in the somatodendritic compartment. Chondroitinase pretreatment completely restored the normalized dendrite growth onto the CSPG stripes (CM = 0.70 vs chABC = 1.06, *p* = 0.001; Fig. 2*E*). Recombinant Reelin addition also increased the amount of dendrite growth onto CSPG-positive stripes, albeit to a lesser extent (CM = 0.70 vs RM = 0.94, *p* = 0.005).

During early cortical development, the MZ is a ∼15-μm region characterized by high CSPG expression (Pearlman and Sheppard, 1996) that is encountered by the growing apical dendrite. We therefore reasoned that it might be more physiologically relevant to measure dendritic growth at the 15-μm edge of the CSPG-positive stripe as opposed to measuring MAP2 signal on the entire CSPG stripe. We took high-magnification images and quantified the amount of MAP2-positive signal in the 15-μm CSPG-positive stripe edge, normalizing to the MAP2-positive signal in the adjacent 15 μm of CSPG-negative stripe (Fig. 2*B–D*). This revealed a greater dendritic inhibitory effect of CSPGs (Fig. 2*F*). Controls showed a dramatic decrease in the ratio of MAP2-positive signal on the CSPG-positive stripe which was rescued by chondroitinase pretreatment (CM = 0.26 vs chABC = 0.97, *p* < 0.001) and partially rescued by Reelin addition (CM = 0.26 vs RM = 0.69, *p* = 0.001). This indicates that dendrites are sensitive to steep changes in CSPG concentration and that Reelin application can counteract the effect of CSPG-mediated dendritic growth inhibition.

### Chondroitinase injection partially rescues *reeler* dendrite growth into the MZ

**Figure 3.**
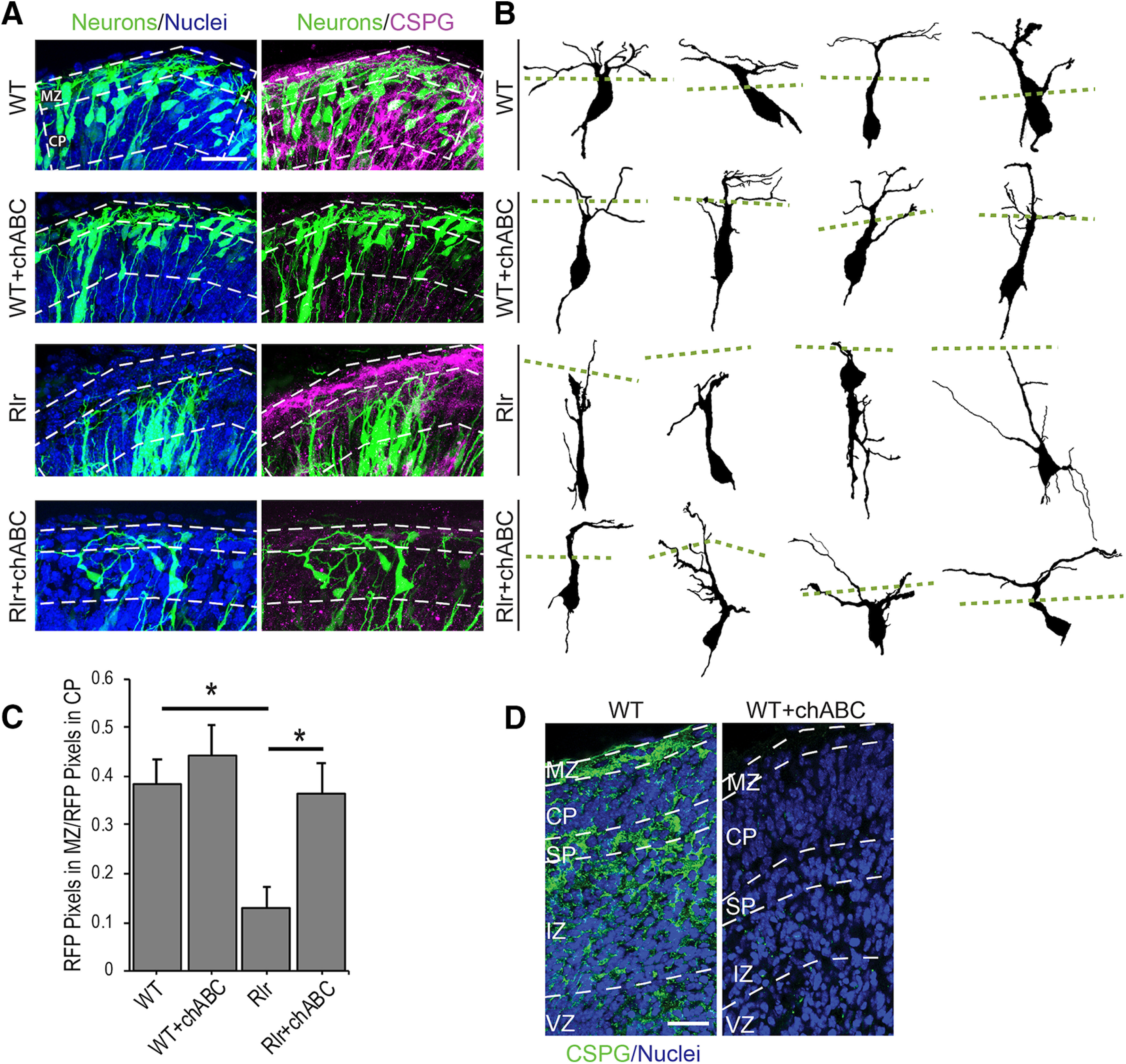
Chondroitinase treatment increases neurite elaboration into the MZ of *reeler* (Rlr) cortices. ***A***, 15-μm z-projections of E15.5 cortical explants transfected with DCX-mRFP (pseudo-colored green) and immunostained with CS56 to label CSPGs (magenta). ***B***, Reconstructed z-projections of representative cells. Green lines represent the beginning of the MZ. chABC was injected into explants at E14.5 and incubated for 16 h. ***C***, Quantification of dendritic growth ratios in *reeler* (Rlr) or *wildtype* (WT). mRFP-positive signal in the MZ was normalized to mRFP-positive signal in the CP. ***D***, Chondroitinase treatment abolished CSPGs confirmed by CS56 immunostaining, without disrupting normal cortical layering architecture. *N* = 12 regions across three explants were quantified for each condition; *, different at *p* < 0.05, one-way ANOVA with Šídák’s multiple comparisons *post hoc* test. Scale bars = 25 μm.

CSPGs localized in the MZ have been suggested to guide axonal tracts of preplate neurons and serve as scaffold for CP formation (Pearlman and Sheppard, 1996), but their role in cortical development is still largely uncharacterized. The observation that dendrites avoid areas of intense CSPG immunoreactivity *in vitro* led us to hypothesize that inactivation of CPSGs might rescue dendrite outgrowth into the MZ in the *reeler*. To test this, we prepared wild-type and *reeler* cortical explants and injected chABC or PBS at multiple points along the dorsal cortex. Explants were electroporated with a DCX-mRFP expression construct to label developing deep layer neurons (Wang et al., 2007). After 1.5 DIV (equivalent to E14.5), the explants were injected with either chondroitinase 100 mU/μl or PBS and then fixed for histologic analyses after an additional 0.5 DIV (Fig. 3*A*). Previous findings showed that *reeler* dendrites display decreased dendrite projection into the MZ, that could be rescued by injection of recombinant Reelin protein in the MZ (O'Dell et al., 2012, 2015). Wild-type neurons displayed a dendritic projection ratio (dendritic mRFP-positive pixels in MZ/mRFP-positive pixels in underlying CP; see Materials and Methods) of ∼0.4 compared with the projection ratio in *reeler* cortices of ∼0.1 (wild type = 0.38 vs *reeler *=* *0.099, *p* = 0.021; Fig. 3*C*), values consistent with prior studies (O'Dell et al., 2012). Importantly, chondroitinase treatment had little effect on dendritic growth (wild-type = 0.38 vs wild-type+chABC = 0.44, *p* = 0.907) and CP formation in wild-type explants (Fig. 3*D*). The efficacy of chondroitinase treatment was confirmed in parallel sections that showed an absence of CS56 immunostaining in chondroitinase-treated areas. Supporting our hypothesis, chondroitinase treatment of *reeler* explants restored the dendritic outgrowth ratio (*reeler *=* *0.099 vs *reeler+*chABC = 0.33, *p* = 0.025; Fig. 3*C*). Although chondroitinase treatment rescued dendrite projections into the MZ, it did not fully revert *reeler* dendrite to wild-type morphology, as the rescued neurons often appeared disoriented and not uniformly apically directed (Fig. 3*B*). This suggests that other factors may contribute to appropriate orientation of cortical neurons (Polleux et al., 2000). In addition, the CSPG core proteins themselves may have residual inhibitory properties, as is the case for versican on axon outgrowth (Schmalfeldt et al., 2000; Dutt et al., 2011).

### Reelin signaling reverses CSPG-induced Akt dephosphorylation

**Figure 4.**
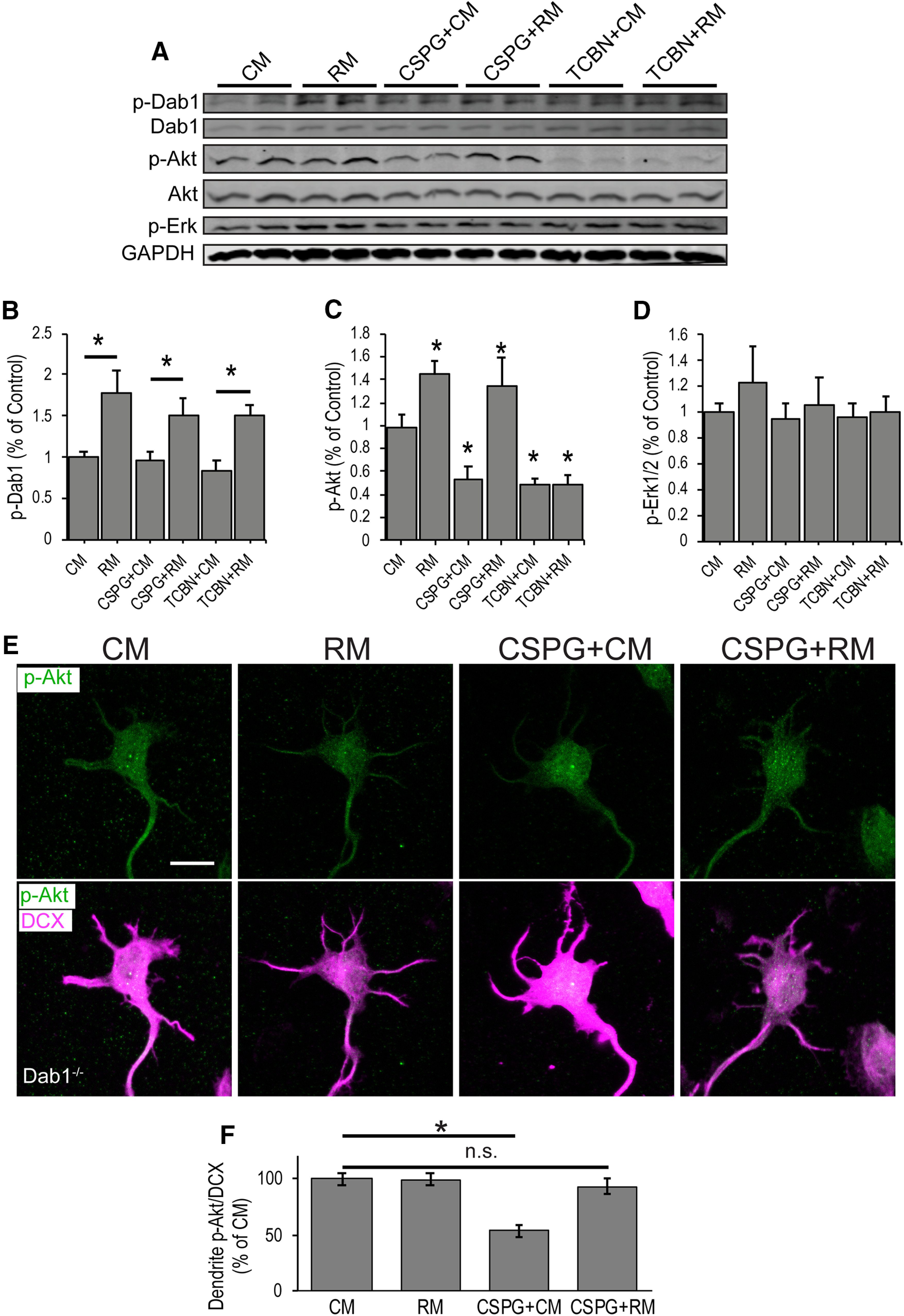
CSPG-induced Akt dephosphorylation is counteracted by Reelin signaling. ***A***, Cortical neurons were cultured for 2 DIV and stimulated for 1 h with CM or RM alone, or in conjunction with CSPGs (3 μg/ml) or Triciribine (TCBN; 1 μm). Western blotting of cortical neuron lysates probed for Reelin signaling proteins. P-Dab1 and Dab1 = ∼80 kDa, p-Akt and Akt = 60 kDa, p-Erk = 44 kDa, GAPDH = 38 kDa. Duplicates of each condition are shown. ***B–D***, Quantification. ***E***, ***F***, Dab1 deficient neurons remain sensitive to CSPG-dependent dephosphorylation but exhibit blunted Akt phosphorylation in response to Reelin stimulation. Cells were immunolabeled with anti p-Akt (S473) and anti-DCX antibodies. p-Akt levels along the dendrites were quantified and normalized to the corresponding DCX signal. *N* ≥ 7 replicates in ***A***, *n* = 25–35 neurons per group in ***E***. Asterisks in ***C*** indicate comparison to CM; *, different at *p* < 0.05, Student’s *t* test in ***C*** and one-way ANOVA with Šídák’s multiple comparisons *post hoc* test in ***F***. Not significant (n.s.). Scale bars = 10 μm.

The Reelin signaling pathway and its role in dendritic growth has been characterized (Pinto Lord and Caviness, 1979; Niu et al., 2004; Olson et al., 2006; Jossin and Goffinet, 2007; Matsuki et al., 2010). Reelin binds to its receptors VLDLR and ApoER2 (Lane-Donovan and Herz, 2017) leading to the activation of Src family kinases (SFKs) and tyrosine phosphorylation of Dab1 (Howell et al., 1999; Bock and Herz, 2003), an essential cytoplasmic adaptor protein (Howell et al., 1997; Sheldon et al., 1997). Reelin signaling diverges to modulate key effectors, including activation of the serine/threonine kinase Akt (protein kinase B) to regulate dendritic outgrowth (Jossin and Goffinet, 2007). Akt is activated by phosphorylation at T308 within the active site (Bozulic and Hemmings, 2009) and S473 within the hydrophobic domain (Hart and Vogt, 2011). Reelin also activates the serine/threonine kinase Erk1/2 through a Dab1-independent pathway to modulate synaptic plasticity (Lee et al., 2014). CSPG signaling also regulates Akt and Erk1/2 activity: CSPGs bind to RPTPσ (Shen et al., 2009) and LAR (Fisher et al., 2011) leading to dephosphorylation of Akt and Erk1/2 in cultured Neuro2A cells and cultured cerebellar granule neurons (Ohtake et al., 2016). Importantly, pharmacological Akt inhibition prevents Reelin-dependent dendritic growth in slice explants (Jossin and Goffinet, 2007). Because of the known opposing biochemical effects on Akt and Erk1/2, and because Reelin application rescued CSPG-induced dendrite inhibition, we hypothesized that Reelin and CSPGs might share antagonistic signaling effectors that are modulated to regulate cortical dendritogenesis. To test whether Reelin and CSPGs have opposing effects on Akt and Erk1/2 phosphorylation state in cortical neurons, we cultured neurons from E15 cortices and stimulated them with soluble neural CSPGs and RM or CM. As expected, Reelin addition led to increased Dab1 and Akt (S473) phosphorylation (CM = 0.98 vs RM = 1.45, *p* = 0.016; Fig. 4*C*), indicative of activation of the canonical Reelin signaling pathway. However, we observed no change in Erk1/2 phosphorylation levels (Fig. 4*D*). CSPG addition led to a decrease in baseline Akt phosphorylation levels (CM = 0.98 vs CSPG+CM = 0.54, *p* = 0.010; Fig. 4*C*) but had no effect on Erk1/2. In support of our hypothesis, Reelin addition reversed CSPG-induced Akt dephosphorylation (CSPG+CM = 0.54 vs CSPG+RM = 1.35, *p* = 0.004). In contrast, pharmacological blockade of Akt activation by Triciribine was not rescued by Reelin addition (TCBN+ CM = 0.49 vs TCBN+RM = 0.48, *p* = 0.970). These findings suggest there may be biochemical convergence of Reelin and CSPG signaling at the level of Akt but not Erk1/2 in cortical neurons.

Because Reelin and CSPGs are both expressed in the extracellular space of the MZ (Nichols and Olson, 2010), it is possible that Reelin and CSPGs interact directly to modulate receptor binding activity (Smock and Meijers, 2018). To first determine whether Reelin-dependent Dab1 phosphorylation occurs in the presence of CSPGs, we examined Dab1 phosphorylation status. While CSPG addition led to a decrease in Akt phosphorylation, Dab1 phosphorylation remained intact (CSPG+CM = 0.95 vs CSPG+RM = 1.51, *p* = 0.042; Fig. 4*B*), suggesting that Reelin and CSPG signals converge intracellularly. To further test this, we cultured neurons from Dab1-deficient (*scrambler*) mice, which do not respond to Reelin, stimulated them with Reelin and CSPGs and measured p-Akt levels by immunostaining with an anti phospho-Akt antibody (Fig. 4*E*). As expected, quantified immunofluorescence signal showed no increase p-Akt levels above baseline after Reelin addition to Dab1-deficient neurons (CM = 100% vs RM = 99.6%, *p* = 0.92; Fig. 4*F*) and CSPG addition caused a decrease in p-Akt in Dab-deficient neurons (CM = 100% vs CSPG+CM = 53.8%, *p* = 0.001). However, the return to baseline p-Akt levels in the CSPG+RM group (CM = 100% vs CSPG+RM = 93.6%, *p* = 0.85) is not expected based on prior Western blotting studies which have demonstrated the necessity for Dab1 in Reelin-dependent Akt phosphorylation. This result warrants further investigation but may indicate that non-canonical (i.e., Dab1-independent) Reelin signaling might be able to return p-Akt levels to baseline after CSPG treatment whereas canonical Reelin signaling may be required to drive p-Akt levels above baseline. Alternatively the residual Dab1 protein in the *scrambler* brain, which has been reported at levels of <5% (Sheldon et al., 1997) may be sufficient to antagonize CSPG signaling but insufficient to drive p-Akt levels above baseline. Taken together, the results indicate that Reelin biochemically counteracts CSPG signaling at the level of p-Akt.

**Figure 5.**
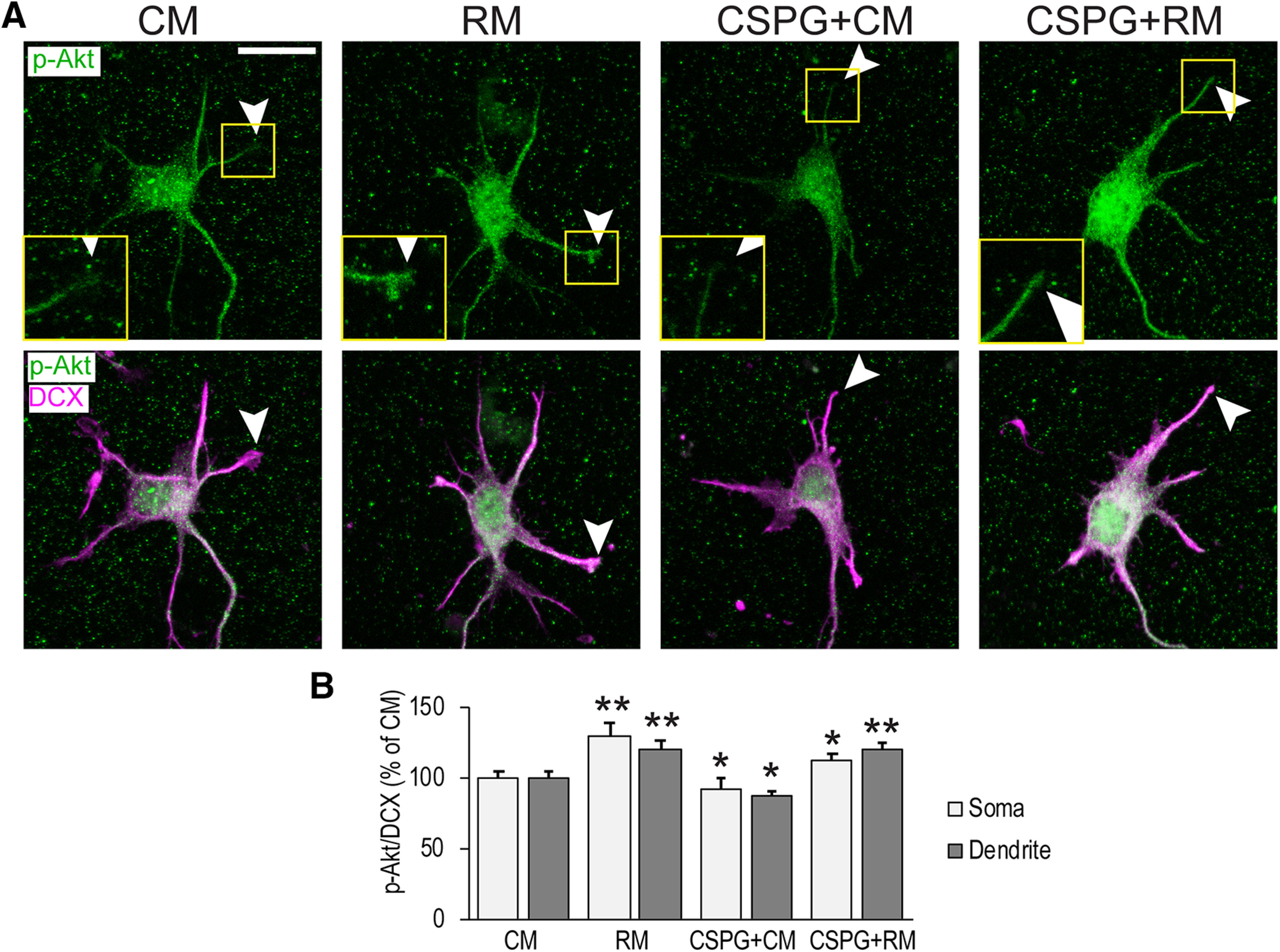
Reelin application counteracts CSPG-induced Akt dephosphorylation in developing neurites. E15 cortical neurons were cultures for 2 d and stimulated with Reelin (RM) or control (CM) media and/or CSPGs. Cells were immunolabeled with anti p-Akt (S473) and anti-DCX antibodies. p-Akt levels along the dendrites and somata were quantified and normalized to the corresponding DCX signal. Arrowheads point to p-Akt signal at dendritic tips; *, different at *p* < 0.05; **, different at *p* < 0.01, one-way ANOVA with Šídák multiple comparisons *post hoc* test. *N* = 30–35 neurons per group. Scale bar = 10 μm.

Because CSPGs and Reelin have concentrated expression in the MZ, where dendrites deploy, we hypothesized that the Reelin/CSPG phospho-Akt response would localize to the nascent dendrite. To test this, we stimulated cultured neurons with CSPGs and Reelin as described above. The phospho-Akt signal was quantified along the length of neuronal dendrites and around the diameter of neuronal somata. p-Akt signal was normalized to the immature marker DCX (Gleeson et al., 1999). In agreement with our previous experiments, CSPG addition led to a decrease in p-Akt levels in the dendrites (CM = 100.00% vs CSPG+CM = 86.7%, *p* = 0.020; Fig. 5*B*), while Reelin increased baseline p-Akt levels (CM = 100.0% vs RM = 120.8%, *p* = 0.008) and reversed CSPG-dependent Akt dephosphorylation (CSPG+CM = 86.7% vs CSPG+RM = 120.4%, *p* = 0.001). In neuronal somata, p-Akt levels were also increased by Reelin addition, and CSPG-induced Akt dephosphorylation was reversed to a slightly lesser degree than in dendrites (CSPG+CM = 92.6% vs CSPG+ RM = 112.0%, *p* = 0.030). These findings support the hypothesis that Reelin signaling and CSPG signaling have opposing effects on a key dendritic outgrowth regulatory pathway in cortical neurons.

## Discussion

The robust, localized expression of CSPGs in the cortical MZ suggests an important role for early dendrite outgrowth. Previous studies of CSPGs and dendrite development have largely focused on brain plasticity, where CSPGs regulate synapse stability and plasticity (Levy et al., 2014). CSPGs are also well known for their role in the axonal development, guiding and restricting axonal process outgrowth (Hartmann and Maurer, 2001). However, the effects of CSPGs on dendrite outgrowth have largely been neglected. This study demonstrates for the first time the cellular and biochemical inhibitory effects that purified CSPGs have on cortical dendrite outgrowth and the role of Reelin signaling in counteracting this inhibition.

Because dendrites normally grow into the CSPG-rich MZ, we expected dendritic processes of cultured neurons to grow freely in the presence of CSPGs. Surprisingly, dendrites were inhibited both by soluble CSPGs and CSPG-positive stripe substrates (Figs. 1, 2). Dendritic avoidance of CSPG sources is reminiscent of dendritic avoidance of the MZ in *reeler* cortices which led us to ask if Reelin addition *in vitro* could rescue dendritic inhibition. Reelin application did rescue dendrite growth on CSPG-positive stripes but to a lesser degree than chondroitinase treatment. Thus, intact CSPGs may maintain some dendritic inhibitory activity *in vitro* that cannot be counteracted by recombinant Reelin application. It does not appear that chondroitinase treatment rescues dendrite inhibition by simply releasing CSPGs from the stripes because we were able to detect CSPG core proteins with an antibody that recognizes chondroitinase insensitive epitopes (Fig. 2). However, chondroitinase treatment does correlate with a depletion of CS56-positive chondroitin sulfate side chains (data not shown), suggesting that CSPG-mediated dendritic inhibition is dependent on intact chondroitin sulfate side chains, either by directly binding to dendritic receptors or anchoring other ECM cofactor(s). The partial recovery of growth onto CSPG-positive stripes after Reelin addition may also reflect the well-established phenomena that neurites will grow on a more preferred substrate when presented with two options (Walter et al., 1987b; Esch et al., 1999). In the stripe assay, the CSPG-positive stripes may remain the less preferred substrate even after Reelin application. Future studies might address this by using neural ECM scaffolds to more closely mimic the 3D extracellular environment (Estrada et al., 2014). Because *reeler* dendrites avoid the MZ, and the stripe assay data suggested a Reelin-CSPG interaction affecting dendrites, we examined dendritic growth ratios in wild-type (with Reelin) and *reeler* (without Reelin) cortical explants treated with and without chondroitinase. If CSPGs inhibit dendrites in the absence of Reelin, then abolishing CSPGs with chondroitinase should rescue dendritic growth in the *reeler* and perhaps to a lesser extent enhance wild-type dendritic outgrowth into the MZ. Indeed, we observed dramatic recovery of dendritic projections into the MZ in *reeler* explants (Fig. 3), supporting the hypothesis that CSPGs themselves or factors closely associated with CSPGs (Paveliev et al., 2016; Djerbal et al., 2017) actively destabilize the nascent dendrite in the absence of Reelin signaling. The observation that chondroitinase treatment did not cause exuberant (excess) dendritic growth in wild-type explants was somewhat surprising but seems to suggest that, normally, Reelin signaling completely counteracts CSPG-mediated inhibition in wild-type cortex. Thus, destruction of CSPGs or the presence of Reelin signaling produces quantitatively the same amount of dendritic growth. In the stripe assay, the low levels of Reelin protein *in vitro* (caused by dissociation and loss of extracellular Reelin protein, coupled with the large volume of media that dilutes any Reelin secreted after dissociation) render the wild-type cultures *reeler*-like (Reelin-deficient). Thus, the chondroitinase-treated cultures model *reeler*+chABC as opposed to wild-type+ chABC. In this case, treatment with chABC, or Reelin, now allows the growth of dendrites onto the stripes.

Neurite projection models suggest that neuronal projections must be coordinated by growth promoting (positive) and growth inhibiting (negative) cues, spatially localized in the target tissue (Gierer, 1981). An example of this is in the optic tectum, where a superficial to deep gradient of Reelin protein is proposed to counteract a superficial to deep negative gradient of Slit proteins to establish neurite targeting to correct synaptic laminae (Di Donato et al., 2018). However, an important consideration is that although CSPGs are broadly considered negative regulators of neurite outgrowth, they have distinct expression patterns, heterogeneous structures and varying effects on neurites (Viapiano and Matthews, 2006; Rauvala et al., 2017). Therefore, isolating specific CSPGs and their isoforms may be necessary to fully characterize their biochemical and cellular effects on dendrite outgrowth. Furthermore, other known or unknown extracellular guidance cues may act in parallel to Reelin and CSPGs to orient the developing dendrite (Polleux et al., 2000). Efforts to identify such molecules might be accomplished by extracting neural ECM for bioassay-guided fractionation (Walter et al., 1990), testing the effects of different neural ECM fractions on dendrite outgrowth.

Although there is a wealth of genetic and biochemical evidence for Reelin’s critical role in cortical development, several key observations have not been reconciled in most models describing Reelin’s cellular function; these gaps need to be addressed to fully understand Reelin’s initial action. Although Reelin promotes Golgi deployment and dendrite outgrowth in hippocampal neurons over several days *in vitro* (Niu et al., 2004; Matsuki et al., 2010), Reelin’s initial, rapid effects on dendrite initiation (O'Dell et al., 2015) have not been recapitulated *in vitro*. It is possible that Reelin’s initial action is to promote cell-cell adhesions between dendrites and Cajal–Retzius cells in the MZ (Gil-Sanz et al., 2013), and *reeler* dendrites lose the ability to make these adhesions. However, high-resolution imaging suggests that <3% of the dendrite surface is in contact with CR cell axons in the MZ, a number similar to the amount of contact expected by random dendritic growth (O'Dell et al., 2015). Furthermore, genetic ablation of the majority of Cajal–Retzius neurons does not lead to a *reeler* phenotype which would be expected in this model (Yang et al., 2000; Yoshida et al., 2006) as CR neurons provide the majority of the cellular elements within the MZ. If Reelin localization is necessary for dendrite initiation and stability, its loss should result in equally unstable dendrites inside and outside of the MZ and/or random neurite orientation. However, imaging of dendritogenesis in explants revealed that *reeler* neurons extend dendrites tangentially, not completely randomly, and avoid the MZ (O'Dell et al., 2012, 2015). Therefore, dendrites prefer to grow into the MZ in the presence of Reelin but avoid the MZ in the absence of Reelin. Taken together, these observations combined with our data suggest that Reelin acts as a signal that stabilizes dendrite growth in the presence of CSPGs.

This study connects the Reelin and CSPG signaling pathways for the first time at the level of Akt. We show that embryonic cortical neurons are sensitive to CSPG-dependent Akt dephosphorylation, which is reversed by Reelin-dependent phosphorylation. We also tested Erk1/2 activity but did not see a phosphorylation response. Akt is a major known contributor to Reelin-dependent dendrite outgrowth during development, while Reelin dependent Erk1/2 signaling functions in postnatal and adult synaptic development (Jossin and Goffinet, 2007; Trotter et al., 2013). Furthermore, Reelin-Erk1/2 signaling predominantly occurs independently of canonical Reelin signaling through VLDLR/ApoER2, suggesting that Erk1/2 activation is not involved the dendritic growth stabilization phenotype (Lee et al., 2014).

**Figure 6.**
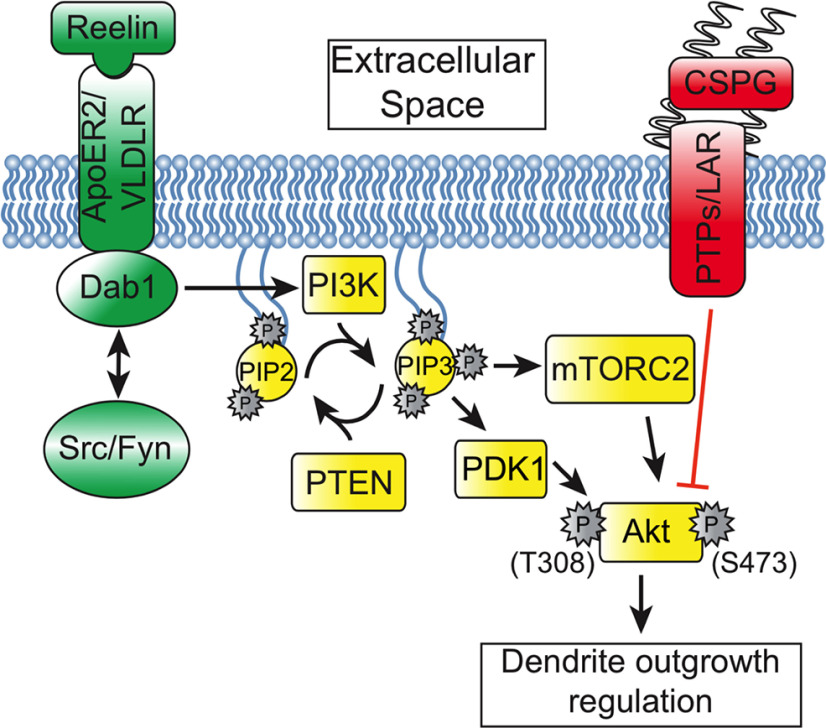
Model of Reelin-Dab1 signaling counteracting CSPG signaling to regulate dendrite growth in the MZ. Reelin and CSPGs are encountered by dendritic filopodia in the MZ. CSPG-mediated dephosphorylation and inactivation of Akt is overcome by Reelin-Dab1 signaling, permitting the outgrowth of a stable dendrite into the MZ. In the *reeler* cortex, CSPG signaling is not counteracted by Reelin-Dab1 signaling and Akt is inactivated, leading to dendrite destabilization.

Future studies should identify the signaling partners that are upstream and downstream of Akt in the Reelin-CSPG relationship. The receptor tyrosine phosphatases RPTPσ and LAR have been shown to mediate CSPG induced neurite inhibition and likely confer neurite inhibition in the context of dendritogenesis (Ohtake et al., 2016), but this needs to be confirmed. Akt has two sites, S473 and T308 that are phosphorylated when Akt is maximally activated and Reelin signaling leads to phosphorylation of both sites (Jossin and Goffinet, 2007). We focused on S473 in this study because it has been reported to be phosphorylated at high levels in the cortical MZ (Itoh et al., 2016) and is implicated in CSPG signaling (Ohtake et al., 2016). In this pathway, Reelin signaling leads to Dab1 recruitment of the p85α subunit of phosphatidylinositol 3 kinase (PI3K). PI3K is a lipid kinase that converts phosphatidylinositol (3,4)-bisphosphate (PIP2) lipids to phosphatidylinositol (3,4,5)-trisphosphate (PIP3; Rameh and Cantley, 1999; Kumar et al., 2005). Akt binds to PIP3 at the plasma membrane where PDK1 triggers phosphorylation of T308 and partial activation of Akt (Bock et al., 2003). Full Akt activation is achieved through subsequent phosphorylation of Akt at S473 by mTORC2 (Manning and Toker, 2017). In addition, mTORC2 might also associate with PI3K and Akt at the plasma membrane (Zoncu et al., 2011). Future studies to determine whether S473 and T308 are differentially activated by Reelin/CSPG stimulation will shed light on this mechanism of dendritic regulation. Precise coordination of cytoskeletal dynamics is certainly critical for dendritic growth modulation at the MZ (Förster, 2014). Indeed, Rho-GTPases regulate cytoskeletal dynamics and are linked to Reelin and CSPG-dependent Akt, PI3K and mTORC2 activity (Jacinto et al., 2004; Leemhuis and Bock, 2011; Ohtake et al., 2016; Tripathi et al., 2017). Given the localized expression of Reelin and CSPGs in the MZ (Nichols and Olson, 2010), along with their associated receptors and signaling machinery in CP neurons (Visel et al., 2004), it is possible that dendrites locally assess Reelin/CSPG signaling to effect cytoskeletal stabilization/destabilization in dendritic filopodia as they extend into the MZ (Fig. 6). The extracellular cues in the MZ are likely key for precise dendritic deployment and subsequent differentiation as well as preventing potential ectopic dendritic growth and inappropriate neural connectivity.

## Synthesis

Reviewing Editor: Deanna Smith, University of South Carolina Decisions are customarily a result of the Reviewing Editor and the peer reviewers coming together and discussing their recommendations until a consensus is reached. When revisions are invited, a fact-based synthesis statement explaining their decision and outlining what is needed to prepare a revision will be listed below. The following reviewer(s) agreed to reveal their identity: Mitsuharu Hattori, Gabriella D’Arcangelo.

Both reviewers agreed this manuscript presents interesting findings that suggest a potential interaction between Reelin and CSPG, and that this interaction, via AKT, serves to control the extent of dendritic growth into the marginal zone. There were concerns about the significance and interpretation of the reported findings, but in general the reviewers agreed that the manuscript is potentially suitable for publication in eNeuro following revision to address their concerns. Please carefully consider the specific comments from each reviewer before resubmitting the manuscript:

Reviewer 1 Comments

In this manuscript, the authors try to show that Reelin counteracts CSPGs in the dendrite formation, particularly in the marginal zone. The experiments are reasonably planned and performed, and the results and discussion are, in general, nicely presented. The downside is that the differences by the treatment with Reelin and/or Chondroitinase in Figures 3-5 are subtle, and thus I am skeptical about the physiological significance of the Reelin/CSPGs interaction. I also noted some errors and unclear points, as stated below.

1. Lines 74-76: the authors write, “During development the neural extracellular matrix (nECM) in the MZ is characterized by high expression levels of chondroitin sulfate proteoglycans (CSPGs) (Aquino et al., 1984; Sheppard et al., 1991).” Aquino et al., (JCB 1984) does not deal with cerebral cortex.

2. Line 214: the authors write, “we dissociated and cultured DCX-mRFP+ neurons from reeler explants 2 days after electroporation...”. In Materials and Methods, they say that Reelin heterozygote mice were mated to produce Reelin-deficient reeler, which means only approximately 1/4 of the embryos are Reelin-deficient. Therefore, I assume that they electroporated 1/4 of the embryos, or they did so all the embryos but used 1/4 of them for explant culture or dissociation. Am I right? I ask this because I think that using reeler neurons is unnecessary in these experiments.

3. Figure 2: please provide the source and cat# of anti-MAP2, anti-SMI312, and Cat-315. How did they perform triple staining?

4. Figure 4: please provide the cat# of anti-Dab1. Did the authors use pY99 antibody for the detection of phosphorylated Dab1?

5. The statistical method used in each experiment is not clear. Please write the method used in the figure legends.

6. Line 290: 100 mu > 100 mU

7. There are many errors in References.

Line 497: no journal name, volume, page numbers.

Line 503: ?? should be corrected.

Lines 523-525: a strange sentence is inserted.

Line 530: is it appropriate to cite 1997a in this context? This paper does not mention Reelin.

Line 535: please provide the last page number.

Line 571: Hardy, J. a. should be Hardy, J.A.

Line 582: please provide details.

Line 589: J. Neurosci. 35O’Dell, 10659-10674. “O’Dell” should be removed.

Line 597: please provide details.

Line 605: Ghosh, a should be Ghosh, A.

Line 612: (pt 5 should be removed.

Line 615: please provide the volume and page numbers.

Line 622: Pearlman, a L. should be Pearlman, A.L.

Line 627: it should be Open Biol.8 180026.

Line 655: please provide the volume and page numbers.

Line 659: Grove, E. a should be Grove, E.A.

Line 662: please provide the volume and page numbers.

The statistical method used in each experiment is not clear. Authors should write the method used in the figure legends.

I think that using One-way ANOVA with Bonferroni post-hoc tests in Figure 1F and 1G is not wrong, but not very appropriate, either. In these panels, the authors put * when p is smaller than 0.008, which I think means that the real p value can be as large as 0.08 (because there are 5 samples, there are 10 (5x4/2) comparisons.) I am not sure this is what authors did because the details of the statistical method are not given.

Reviewer 2 Comments

In this manuscript the authors investigate the mechanisms controlling dendrite outgrowth of cortical neurons into the marginal zone. They demonstrate that CSPG inhibits it and that Reelin counteracts it, thus enabling growth in WT but not reeler mice. The signaling pathways are proposed to converge at the level of Akt. Overall, the data is novel and interesting. However, some issues should be addressed:

- Fig. 1. The data indicate that Reln can rescue the CSPG effect on dendrite growth, but not axons. It is unclear how these processes are defined since they are both labeled by RFP.

- Fig. 2. Related to the above, many processes co-label with dendrite and axonal markers. It seems more appropriate to refer to these as neurites rather than trying to distinguish between dendrite and axons, which is not really possible in vitro. Also, MAP2+ cell bodies are clearly seen entering the CSPG stripes after treatment with either Reelin or chABC - are these included in the MAP2+ signal plotted in E and F? Regardless, the data show that chABC treatment (like Reelin) facilitates the outgrowth of WT MAP2+ processes.

- Fig. 3. Contrary to the previous results, these explant data indicate that chABC treatment of WT neurons has no effect on their ability to enter the MZ. The authors need to reconcile or discuss these findings. On the other hand, chABC treatment of reeler neurons completely rescues their growth defect in the MZ. This is a remarkable finding - in light of the model proposed here, it would be very interesting to understand how chABC treatment affects Akt phosphorylation in the explants.

- Fig. 4. The Western blot data convincingly show that CSPG suppresses Akt phosphorylation whereas Reelin upregulates it and rescues it. But the immunofluorescence data in Dab1 KO neurons is confusing. It seems that Reelin rescues the CSPG-induced defect in mutant neurons. How can this be? It has been shown multiple times that Dab1 is required to mediate Akt phosphorylation by Reelin in the canonical pathway...

- Fig. 5. These pAkt immunofluorescence data confirm that Reelin counters the CSPG inhibition in WT neurons (dendrites and soma). No problems here.

- Fig. 6. The model points to Akt as the convergence point between the 2 pathways. Most data (except the Dab1 KO data in Fig. 4) support the model. A minor inaccuracy is the positioning of the Akt phospho-sites; the arrow from PDK1 should point to the T308 site (direct target), and the one from mTORC2 to the Ser473.

- Statistics. The use of the asterisks in the plots is very confusing. It is more common to use an appropriate number of asterisks to indicate the degree of confidence, for example *p<0.05, **p<0.001, ***p<0.0001. Also, it would be good to clearly indicate what the n values represent in each plot, and to state the software used.

- Minor typos: ‘that that’ on line 235. ‘H-I’ on line 671.

The use of asterisks for p values should be corrected
